# Adenosine deaminase production by an endophytic bacterium (*Lysinibacillus* sp.) from *Avicennia marina*

**DOI:** 10.1007/s13205-013-0144-2

**Published:** 2013-06-07

**Authors:** Kandasamy Kathiresan, Kandasamy Saravanakumar, Sunil Kumar Sahu, Muthu Sivasankaran

**Affiliations:** Faculty of Marine Sciences, Centre of Advanced Study in Marine Biology, Annamalai University, Parangipettai, 608 502 Tamil Nadu India

**Keywords:** Mangroves, Endophytic bacteria, Adenosine deaminase and 16S rRNA

## Abstract

The present study was carried out with the following objectives: (1) to isolate the endophytic bacilli strains from the leaves of mangrove plant *Avicennia marina,* (2) to screen the potential strains for the production of adenosine deaminase, (3) to statistically optimize the factors that influence the enzyme activity in the potent strain, and (4) to identify the potent strain using 16S rRNA sequence and construct its phylogenetic tree. The bacterial strains isolated from the fresh leaves of a mangrove *A. marina* were assessed for adenosine deaminase activity by plating method. Optimization of reaction process was carried out using response surface methodology of central composite design. The potent strain was identified based on 16S rRNA sequencing and phylogeny. Of five endophytic strains, EMLK1 showed a significant deaminase activity over other four strains. The conditions for maximum activity of the isolated adenosine deaminase are described. The potent strain EMLK1 was identified as *Lysinibacillus* sp. (JQ710723) being the first report as a mangrove endophyte. Mangrove-derived endophytic *bacillus* strain *Lysinibacillus* sp. EMLK1 is proved to be a promising source for the production of adenosine deaminase and this enzyme deserves further studies for purification and its application in disease diagnosis.

## Introduction

Endophytic bacteria are important source for developing the novel drugs for effective treatment of diseases in humans, plants and animals (Strobel et al. 2004). The mangroves do have colonized endophytic bacteria, but the potential of the bacteria for medicinal enzymes are largely unexplored (Gayathri et al. 2010). Adenosine deaminase-ADA (EC 3.5.4.4) is a zinc-metallo enzyme involved in purine metabolism (Alrokayan 2002, 2007). This enzyme is widely distributed in different species (Bachrach 2004; Pospisilova 2007) but not reported from mangrove endophytes. The genes encoding these deaminases are essential in bacteria and yeast (Losey et al. 2006). This enzyme in particular is of special interest for its role in cellular growth regulation and differentiation (Hershfield and Mitchell 1995). Two types of the adenosine deaminase occur in human as ADA1 and ADA2; Lymphocytes and macrophages cells are known to have the ADA1 (Hirschhorn and Ratech 1980; Srinivasa Rao et al. 2010). Clinical and in vitro studies strongly suggest a mutual relationship between the absence of adenosine enzymatic activity and the immunodeficiency disease (Booth et al. 2006), which is characterized by severe defects in cellular and humoral immunity (Jasmin et al. 2012). The production of adenosine deaminase in the marine environment in particular mangrove biotope is almost non-existent. Hence, the present study was attempted to isolate endophytic bacilli from the mangrove plant *A. marina* for exploring the production of adenosine deaminase and to identify the potent strain by using 16S rRNA sequencing.

## Materials and methods

### Sample collection and surface sterilization of leaves

Healthy mangrove leaves of *A. marina* (Forsk.) Vierh were collected from Vellar estuary, Parangipettai, Tamil Nadu, India. All the samples were collected in sterile plastic bags and transported aseptically to the laboratory. The leaves were washed in running tap water, followed by 70 % ethanol for 2 min, 2 % sodium hypochlorite containing 0.1 % Tween 20 for 10 s and finally rinsed in distilled water for 2 min.

### Isolation of endophytic *Bacillus* sp.

The washed leaves were crushed in a mortar and pestle. About 1 mL of crushed sample was serially diluted up to 10^−5^ dilutions using 12.5 mM potassium phosphate buffer (pH 7.1). For the isolation of endophytic bacillus, 0.1 mL of aliquot from 10^−2^ to 10^−5^ dilutions was inoculated by spread plate method on MRS (de man rogosa sharps) agar medium using sterile L‐rod in Petri plate. The plates were incubated at 28 °C for 120 h.

### Purification and selection of endophytic bacillus

Morphologically different bacterial colonies were selected and streaked on nutrient agar plates and incubated at 28 °C for 48 h. Five morphologically different colonies were purified by continuous cluttering method and the purified bacterial strains were named as EMLK1, EMLK2, EMLK3, EMLK4 and EMLK5. All the selected isolates were subcultured in nutrient agar slants and preserved in refrigerator at 4 °C.

### Confirmation of adenosine deaminase activity in *Bacillus* strains

Production of the enzyme was confirmed by the change in colour from yellow to pale red by spectrophotometer which is due to the release of ammonia by increase in the pH level on modified Czapek Dox agar medium (Eaton et al. 1998; Shanmugam 2011) inoculated with the five *Bacillus* strains separately.

### DNA extraction

The modified and standardized method of Sambrook and Russell (2001) was used to extract DNA from broth culture of EMLK1 strain. Briefly, 2 mL of broth culture of the sample in log phase was taken in Eppendorf tube. The tube was spun at 12,000 rpm for 10 min. The supernatant was discarded and the pellet dissolved completely in 500 μL of lysis buffer solution followed by addition of 10 % SDS and 5 μL proteinase K and incubated for 1 h at 60 °C. 250 μL of 5 M NaCl was added and chilled on ice for 10 min. Centrifugation was carried out at 8,000 rpm for 15 min and the supernatant was transferred to 1.5 mL Eppendorf tube. DNA was precipitated by adding chilled absolute ethanol and inverted gently several times. Then it was incubated at −20 °C for 3–4 h followed by centrifugation at 13,000 rpm for 15 min. Supernatant was discarded and the pellet was washed with 70 % ethanol. Pellet was air dried at room temperature and resuspended in 50 μL of TE buffer (pH 8.0) for further use.

### 16s rRNA amplification and sequencing

16s rRNA amplification reaction was performed using 16S primers 27F and 1492R in a 0.2 mL optical-grade PCR tube (Tarsons, India). 50 ng of DNA extract was added to a final volume of 50 μL of PCR reaction mixture containing 1.5 mM MgCl_2_, 1× Reaction buffer (without MgCl_2_) (Fermentas), 200 μM of each dNTPs (Fermentas), 100 pM of each primer and 1.5 U Taq DNA polymerase (Fermentas). PCR was performed in an automated thermal cycler (Lark Research Model L125+, India) with an initial denaturation at 95 °C for 5 min followed by 30 cycles of 95 °C for 30 s (denaturation), 52 °C for 45 s (annealing), 72 °C for 90 s (extension) and 72 °C for 10 min (final extension). PCR product was run on 1 % agarose in TAE buffer (40 mM Tris, 20 mM Acetic acid, 1 mM EDTA [pH 8.0]) to confirm that the right product (1,500 bp) was formed. The PCR product was purified using the QIAGEN PCR purification kit for sequencing. DNA sequencing was carried out using 3730 Genetic analyser, Applied biosystems, USA (Ramachandra Innovis, Chennai, India).

### Phylogenetic analyses

Sequence similarity search for the obtained sequence was performed against the non-redundant database maintained by the National Center for Biotechnology Information using the BLAST algorithm (http://www.ncbi.nlm.nih.gov). Then the 16S rRNA sequence of the isolate was aligned with the sequences of selected reference taxa from NCBI and Ribosomal Database Project II (http://rdp.cme.msu.edu) using the ClustalW implemented in the MEGA5 software (Tamura et al. 2011) and the alignment was inspected and adjusted manually where necessary. The aligned sequences were incorporated to construct phylogenetic tree using maximum-likelihood method (Saitou and Nei 1987). All characters were equally weighted and unordered. Initial tree(s) for the heuristic search were obtained automatically by applying Neighbour-Join and BioNJ algorithms to a matrix of pairwise distances estimated using the maximum composite likelihood approach, and then selecting the topology with superior log likelihood value. Alignment gaps were treated as missing data. MP analysis was conducted using a heuristic search. *Bacillus subtilis* (JN609214) was taken as outgroup taxon. The robustness of trees in the maximum-likelihood (ML) analyses was evaluated by 1,000 bootstrap replications.

### Fermentation and optimization of reaction condition for deaminase activity

The optimization of the reaction conditions for deaminase activity was analyzed in fermentation medium containing folic acid (0.5 g), dextrose solution (4.5 g) and mineral solution (133 %) which constituted of NH_4_NO_3_ (0.213 g), MgSO_4_ (0.027 g), ZnSO_4_ (0.001 g), CaCl_2_ (0.007 g), MnSO_4_ (0.003 g), CuSO_4_ (0.001 g), FeSO_4_ (0.013 g), NaSO_4_ (0.027 g) and NaH_2_PO_4_ (0.027 g). The response surface methodology was used to statistically optimize the important reaction factors: pH in the range of 4.0–8.0, reaction time (0–30 min), concentrations of adenosine (10–50 μL), and concentrations of microbial cell free culture filtrate (10–50 μL).

### Statistical optimization

Deaminase activity was optimized using a standard response surface methodology design also known as central composite design (CCD). The range and the levels of the variables (high and low) considered in the present work are given in Table 1. Adenosine deaminase activity (U L^−1^) (Y) was taken as the response of the design experiments. The quadratic equation model for predicting the optimal point is expressed according to Eq. 1.

**Table 1:** Experimental range and levels of independent process variables

Factor	Range and coded value				
	−2	−1	0	1	2
pH	4	5	6	7	8
Reaction time (min)	0	10	20	30	40
Adenosine concentration (μL)	10	20	30	40	50
Microbial cell free culture filtrate concentration (10–50 μL)	10	20	30	40	50

1 $$\begin{array}{c} Y\;=\beta_{0}+\;\beta_{1}X_{1}+\;\beta_{2}X_{2}+\;\beta_{3}X_{3}+\;\beta_{4}X_{4}+\;\beta_{11}X_{1}^{2}+\;\beta_{22}X_{2}^{2}+\;\beta_{33}X_{3}^{2}+\;\beta_{44}X_{4}^{2}+\;\beta_{12}X_{1}X_{2}+\\ \beta_{13}X_{1}X_{3}+\;\beta_{14}X_{1}X_{4}+\;\beta_{23}X_{2}X_{3}+\;\beta_{24}X_{2}X_{4}+\;\beta_{34}X_{3}X_{4} \end{array}$$

where *X*_1_ is pH (°C), *X*_2_ is reaction time (min), *X*_3_ is adenosine concentration (μL), *X*_4_ is microbial cell free culture filtrate (μL).

Four factors were studied and their low and high levels of actual values. Thirty-two experiments were conducted in duplicate. Design Expert Version 8.0.6 (Stat Ease, USA) was used for data analysis. The optimum values of the selected factors were obtained by fitting the regression equation and by analyzing the contour and surface plots. The multiple coefficient of determination was used and variability among dependent variables was explained. *R*^2^ and the model equation were used to predict the optimum value and subsequently the interaction between the factors within the specified range was elucidated (Elibol and Ozer 2002). The adenosine deaminase activity was calculated by following the method of the (Giusti and Galanti 1984). Their mean values and 5 % standard errors were calculated.

## Results

**Fig. 1:**
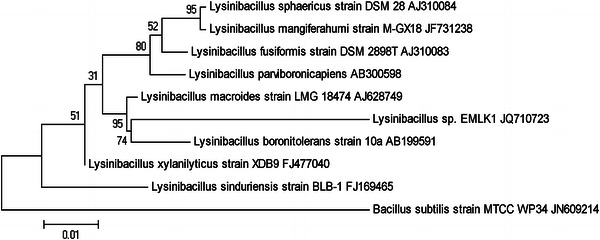
Molecular phylogenetic analysis of *Lysinibacillus* sp. by maximum likelihood method

The surface sterilized leave samples of *A. marina* were subjected for the isolation of endophytic *Bacillus* sp. and tested for the adenosine deaminase enzyme activity. Among the selected five strains only the *Bacillus* strain EMLK1 showed the significant enzyme activity as revealed by zone of inhibition. 16S rRNA sequencing result revealed that the potential strain was *Lysinibacillus* sp. (Genbank accession number: JQ710723). The evolutionary history was inferred by using the Maximum Likelihood method based on the Tamura–Nei model (1993). The tree with the highest log likelihood (−2,548.3764) is shown in Fig. 1. The percentage of trees in which the associated taxa clustered together is shown next to the branches. The tree is drawn to scale, with branch lengths measured in the number of substitutions per site. The analysis involved 10 nucleotide sequences. All positions containing gaps and missing data were eliminated. There were a total of 1,058 positions in the final dataset. Evolutionary analyses were conducted in MEGA5 (Tamura et al. 2007).

Recently, literature data compiled from hundreds of species descriptions has suggested that strains sharing less than 98.8 % sequence similarity belong to different genospecies. Therefore, additional DNA–DNA hybridization study is required to differentiate the identified *Lysinibacillus* strain up to species level more accurately.

The experimental model fitness for deaminase activity was tested by the quadratic model along with the contour error plot and normal probability plot and the ADA activity for each cycle performed as per the experimental design along with experimental response and predicted response. The response surface methodology based on the estimates of the parameters indicated an experimental relationship between the response and input variables expressed by the following quadratic model Eq. (2).

2 $$\begin{array}{c} \text{ADA activity}=8.50\;+\;0.95A\;+\;2.23B\;+\;1.08C\;+\;0.74D\;-\;0.32\mathrm{AB}\;+\;0.33\mathrm{AC}\;+\;0.58\mathrm{AD}\;+\;\\ \;0.76\mathrm{BC}-0.026\mathrm{BD}+1.18\mathrm{CD} \end{array}$$

where *A*, *B*, *C* and *D* are independent variables. Significance of each coefficient presented in Eq. (2) was determined by the student’s *t* test and *p*-values. The results of the quadratic model for the enzyme activity are in the form of analysis of variance (ANOVA). The value of predicted *R*^2^ and adjusted *R*^2^ is close to 0.92 revealing a high correlation between the observed values and the predicted values. The means that regression model provides an excellent explanation of the relationship between the independent variables (factors) and the response (enzyme activity). The lack-of-fit term was non-significant as it was desired. The non-significant value (0.76) of lack-of-fit observed was more than probability of 0.05 which revealed that the quadratic model was valid for the present study. The statistical optimization revealed the optimized conditions of reaction: pH 6.59, 31.89 min of reaction time, 47.84 μL of adenosine concentration and 40.27 μL of microbial cell free culture filtrate for the better production of the adenosine deaminase enzyme activity.

## Discussion

Nowadays, the study of adenine/adenosine deaminases is receiving much attention due to diseases that are induced in the absence or excess of the enzyme activity. The production of the enzyme has been reported in several microbes: *Escherichia coli* (Sugadev et al. 2006), *Enterococcus faecalis* (Pospisilova et al. 2006) *Saccharomyces cerevisiae* and *Schizosaccharomyces pombe* (Lee et al. 2009). Although bacterial bacilli are reportedly producing adenosine deaminase (Jun et al. 1991), the present study reported for the first time the enzyme production by endophytic bacillus, *Lysinibacillus* sp. derived from mangroves. This enzyme activity could be due to the presence of pterin synthesizing biochemical compounds in *Lysinibacillus* sp.

The statistically optimized conditions for enzyme activity in the present study are in accordance with the earlier reports of Sakai and Jun (1978) who reported that optimum pH for the reaction was 5.0–6.0, and the optimum temperature was 55 °C for production of extracellular adenosine deaminase by the endophytic *Streptomyces* sp. The enzyme in *Lysinibacillus* sp. was found to be unstable, but protected from inactivation by ethyl alcohol and this result is in agreement with the results of Sakai and Jun (1978).

## References

[CR1] Alrokayan SAH (2002). Purification and characterization of adenosine deaminase from camel skeletal muscle. Int J Biochem Cell Biol.

[CR2] Alrokayan SAH (2007). Role of adenosine deaminase and purine nucleoside phosphorylase in severe combined immunodeficiency disease: a biochemical and molecular study. Biosci Biotechnol Res Asia.

[CR3] Bachrach U (2004). Polyamines and cancer: minireview article. Amino Acids.

[CR4] Booth C, Hershfield M, Notarangelo L, Buckley R, Hoenig M, Mahlaoui N (2006). Management options for adenosine deaminase deficiency: proceedings of the EBMT satellite workshop. Clin Immunol.

[CR5] Eaton AD, Clesceri LS, Greenberg AE (1998). Standard methods for the examination of water and waste water.

[CR6] Elibol M, Ozer D (2002). Response surface analysis of lipase production by freely suspended *Rhizopus arrhizus*. Process Biochem.

[CR7] Gayathri S, Saravanan D, Radhakrishnan M, Balagurunathan R, Kathiresan K (2010). Bio prospecting potential of fast growing endophytic bacteria from leaves of mangroves and salt marsh plants species. Indian J Biotechnol.

[CR8] Giusti G, Galanti B, Bergmeyer HU (1984). Colorimetric method. Methods of enzyme analysis.

[CR9] Hershfield M, Mitchell B, Scriver C (1995). Immunodeficiency diseases caused by adenosine deaminase deficiency and purine nucleoside phosphorylase deficiency. The metabolic and molecular basis of inherited disease.

[CR10] Hirschhorn R, Ratech H (1980) Isonzymes of adenosine deaminase. In: Rattazzi MC, Scandalios JG, Whitt GS (eds) Isoenzymes: current topics in biological and medical research, vol 4. Alan R Liss, Inc, New York, pp 131–157

[CR11] Jasmin HJ, Kaushik VB, Anand BV, Vaidehi RP, Sankalp MS, Dipmala P (2012). Value of adenosine deaminase level for the differential diagnosis various meningitis. Int J Biol Med Res.

[CR12] Jun HK, Kim TS, Sakai T (1991). Purification and characterization of extracellular adenosine deaminase from *Streptomyces* sp. J Ferment Bioeng.

[CR13] Lee G, Lee SS, Kay KY, Kim D, Choi S, Jun KH (2009). Isolation and characterization of a novel adenosine deaminase inhibitor, IADA-7, from *Bacillus* sp. J-89. J Enzyme Inhib Med Chem.

[CR14] Losey HC, Ruthenburg AJ, Verdine GL (2006). Crystal structure of *Staphylococcus aureus* tRNA adenosine deaminase TadA in complex with RNA. Nature Struct Mol Biol.

[CR15] Pospisilova H, Frebort I (2007). Aminohydrolases acting on adenine, adenosine and their derivatives. Biomed Pap Med Fac Univ Palacky Olomouc Czech Repub.

[CR16] Pospisilova H, Novak O, Frebortova J, Strnad M, Frebort I (2006) Oxidative and hydrolytic cleavage of cytokinin derivatives with biomedical and biotechnological potential. In: Abstracts of International Symposium of Fifth 21st Century COE “Towards Creating New Industries Based on Inter-Nanoscience”. Awaji, Japan, pp 15–20

[CR18] Saitou N, Nei M (1987). The neighbor-joining method: a new method for reconstructing phylogenetic trees. Mol Biol Evol.

[CR17] Sakai T, Jun H (1978). Purification and crystallization of adenosine deaminase in *Pseudomonas iodinum*. FEBS Lett.

[CR19] Sambrook J, Russel DW (2001). Rapid isolation of yeast DNA. Molecular cloning, a laboratory manual.

[CR20] Shanmugam S (2011). Optimization of synergistic parameters for atypical pterin deaminase activity from rattus norvegicus using response surface methodology Biochemical and Molecular Engineering XVII.

[CR21] Srinivasa Rao K, Anand Kumar H, Rudresh BM, Srinivas T, Harish Bhat K (2010). A Comparative study and evaluation of serum adenosine deaminase activity in the diagnosis of pulmonary tuberculosis. Biomed Res.

[CR22] Strobel G, Daisy B, Castillo U, Harper J (2004). Natural products from endophytic microorganisms. J Nat Prod.

[CR23] Sugadev R, Kumaran D, Burley SK, Swaminathan S (2006). Crystal structure of an adenine deaminase. New York Structural Genomics Research Consortium (NYSGRC).

[CR24] Tamura K, Nei M (1993). Estimation of the number of nucleotide substitutions in the control region of mitochondrial DNA in humans and chimpanzees. Mol Biol Evol.

[CR25] Tamura K, Dudley J, Nei M, Kumar S (2007). MEGA4: molecular evolutionary genetics analysis (MEGA) software version 4.0. Mol Biol Evol.

[CR26] Tamura K, Peterson D, Peterson N, Steker G, Nei M, Kumar S (2011). MEGA5: molecular evolutionary genetics analysis using maximum likelihood, evolutionary distance, and maximum parsimony methods. Mol Biol Evol.

